# Loneliness in adolescents and youth with bipolar disorder: a scoping review

**DOI:** 10.3389/fpsyt.2026.1696515

**Published:** 2026-03-13

**Authors:** Aroldo Dargél, Tanya Tanya, Caitlin Morris, Risa Shorr, Kathleen Pajer

**Affiliations:** 1 Faculty of Medicine, Department of Psychiatry, University of Ottawa, Ottawa, ON, Canada; 2 Precision Psychiatry and Artificial Intelligence for Early Intervention and System Innovation in Affective Disorders (PRECISAI lab), Department of Psychiatry, University of Ottawa, Ottawa, ON, Canada; 3 Department of Mental Health, The Ottawa Hospital, Ottawa, ON, Canada; 4 Neuroscience Program, Ottawa Hospital Research Institute (OHRI), Ottawa, ON, Canada; 5 Department of Psychiatry, University of Ottawa, Children’s Hospital Eastern Ontario (CHEO) Research Institute, Ottawa, ON, Canada; 6 Department of Life Sciences, Queen’s University, Kingston, ON, Canada

**Keywords:** adolescents, allostatic load, bipolar disorder, loneliness, mood symptoms, psychological stress, social isolation, youth

## Abstract

**Introduction:**

Bipolar disorder (BD) commonly emerges in adolescence, a period marked by heightened mood lability and shifting social roles that may amplify interpersonal stressors and functional risk. Loneliness and social isolation are transdiagnostic, developmentally salient constructs associated with psychiatric morbidity and physiological dysregulation, yet their relevance to youth BD has not been comprehensively summarized. This review synthesizes evidence on loneliness and social isolation in adolescents and youth with BD.

**Methods:**

We systematically searched PubMed, PsycINFO, Embase, and CINAHL from inception to July 2025 using terms related to BD, loneliness/social isolation, and adolescents/youth. Peer-reviewed studies were eligible (no language/date restrictions) if they included participants with a formal diagnosis of BD and measured loneliness and/or social isolation. Of 514 records screened, six studies met inclusion criteria. Methods followed the PCC framework, JBI Reviewer’s Manual, and PRISMA-ScR guidelines.

**Results:**

Six studies (N = 522) included adolescents and young adults with BD. Across heterogeneous designs, loneliness and/or social isolation were repeatedly associated with BD-relevant outcomes, including diagnostic group differences, altered neural responses to social exclusion, and temporal proximity to mood episodes. Social isolation was generally related to poorer psychosocial functioning.

**Conclusion:**

Available evidence suggests that loneliness and social isolation are meaningfully related to clinical and functional correlates in youth BD and may help distinguish BD from comparison groups, although causal and temporal inferences remain limited. Longitudinal and mechanistic studies are needed to clarify directionality and pathways (e.g., mood, cognition, stress biology). Clinically, routine assessment and interventions targeting loneliness/social isolation may be warranted.

## 1 Introduction

Bipolar disorder (BD) is a major public health concern, associated with premature mortality and elevated risk for aging-related conditions such as hypertension, diabetes, and dementia (1–3). The risk of suicide is 20–30 times higher in BD than in the general population and among the highest of all psychiatric disorders (2). Diagnosis and management remain challenging, particularly in youth, due to the absence of biomarkers and reliance on subjective information from patient’s self-report and clinician’s observation (2, 4). Misdiagnosis and delayed treatment can prolong illness, hinder mood stabilization, and contribute to behavioral (e.g., emotional dysregulation), systemic (e.g., cardiometabolic disturbances), and functional (e.g., cognitive decline) consequences (2, 3, 5, 6).

BD most often emerges during adolescence, a developmental period marked by ongoing social and emotional maturation (4, 7). During this sensitive window, early difficulties in emotion regulation—such as mood lability, anger outbursts, and irritability—often precede diagnosis (7–9) and may disrupt peer relationships and social connectedness. Consistent with the dynamic interplay between BD and loneliness, youth with BD commonly experience heightened loneliness/social isolation (10–12), poor social support, and interpersonal difficulties (13–16), which both signal illness-related social and affective difficulties and exacerbate emotional dysregulation, relapse risk, and suicidality (17, 18). Moreover, prodromal symptoms such as anxiety, depression, irritability, and impulsivity increase vulnerability to BD spectrum disorders (4, 8, 10, 11), while loneliness and social isolation are associated with more severe illness trajectories (13, 15, 19, 20). Together, these findings underscore a reciprocal process in which BD-related social-emotional challenges contribute to loneliness, which in turn amplifies illness progression and functional impairment. These vulnerabilities highlight the need for early identification and developmentally tailored interventions to prevent onset or relapse, attenuate progression, and enhance functional recovery in youth with BD.

Loneliness-defined as the subjective perception of inadequate or unfulfilling social connections rather than the objective number of relationships (21–24), is increasingly recognized as a transdiagnostic construct that both co-occurs with and predicts psychiatric illness (25, 26). For instances, loneliness predicts anxiety onset and hinders depression recovery, with links across mood, anxiety, and substance-use disorders (26, 27). Within the context of BD, loneliness and social isolation may represent not only a symptom but also potential risk factors (28), and have been associated with greater mood instability and psychosocial impairment.

Adolescents and young adults are especially vulnerable to loneliness due to ongoing developmental and social transitions, during which peer relationships play a key role in identity formation (29, 30). Disruptions to these interactions—such as exclusion or rejection—can foster persistent loneliness (10, 19, 30), which has been linked to addictive behaviors (20, 31, 32) and increased vulnerability to psychiatric disorders (15, 26, 27). The COVID-19 pandemic intensified these effects: over half of individuals aged 16–24 reported loneliness—compared to 30.9% in the general population—alongside higher levels of anger and perceived stress (33). Chronic loneliness predicts youth depression (16, 34), reduces quality of life, and is compounded by stigma surrounding help-seeking (14, 30). Beyond psychological effects, social isolation during adolescence has been associated with long-term physiological consequences, including cardiometabolic and inflammatory disturbances (24, 35–38). Genetic studies further reveal shared heritability between loneliness and depression—especially pronounced in youth—as well as distinct genetic loci linked to loneliness (32, 39), suggesting overlapping biological pathways connecting loneliness, mood disorders, and cardiovascular risk factors (39, 40).

Beyond its developmental and psychiatric implications, loneliness is increasingly recognized as a global public health issue (41). The World Health Organization (WHO) estimates that 20% of adolescents and one in four elderly people experience loneliness (21), with major economic costs —for example, £3.14 billion annually in the United Kingdom due to lost productivity) (21). Loneliness is also associated with a 30% increased risk of cardiovascular disease, dementia, and premature mortality (42, 43). Chronic loneliness exerts profound neurobiological and behavioral effects, perpetuating a cycle of hypervigilance to social threat, hostile responses, and further social withdrawal (27, 37). Stress from loneliness or social isolation can trigger hypothalamic–pituitary–adrenal (HPA) axis hyperactivity, immunometabolic and mitochondrial dysfunction (44–47). Functional MRI studies demonstrate that lonely individuals allocate greater attention to negative social interactions and derive less reward from positive ones, reinforcing negative cognitive biases (12, 48, 49). Over time, loneliness increases vulnerability to both metabolic and psychiatric disorders through heightened stress sensitivity (44–46), impaired executive function (50), poor sleep (51, 52), and maladaptive behaviors (38, 53–55).

Despite this growing body of evidence, most existing reviews have focused on elderly (26, 56) or non-clinical adult populations (41), overlooking the distinct impact of loneliness on youth. Although BD and loneliness are each common in youth (20, 30, 33), their co-occurrence and clinical relevance remain insufficiently characterized. To address this gap, this scoping review maps the existing literature on loneliness and/or social isolation in adolescents and young adults with BD and summarizes reported associations with illness- and functioning-related outcomes.

## 2 Materials and methods

### 2.1 Search strategy

The search was conducted in four electronic databases- PubMed, PsycINFO, EMBASE, and CINAHL - from their inception to July 2025, using a combination of the following search terms bipolar disorder (e.g., mania), youth (e.g., adolescent), and loneliness (e.g., social exclusion) (see **Supplementary Material** for detailed search strategy). To ensure a comprehensive understanding of social experiences relevant to youth with BD, we expanded our search scope to include both loneliness and social isolation. Conceptually, loneliness refers to the *subjective and distressing experience* of feeling socially disconnected or inadequately connected to others, even in the presence of social contact (23, 57). In contrast, social isolation is an *objective state* characterized by limited social contact or participation in social activities. Although conceptually distinct, social isolation is closely linked to loneliness and is often used as a loneliness proxy in both clinical and research contexts, particularly when subjective measures of loneliness are unavailable or not reported (22, 23, 58). The search strategy was developed in collaboration with a librarian (RS) and the reference lists from identified studies were also hand searched for any additional publication. The protocol for this study was published on OSF in August 2025 (59).

All the selected articles were peer-reviewed primary research publications. No restrictions were applied to the publication date or language. After conducting the search and importing articles into Covidence, we applied the inclusion and exclusion criteria to select studies for the review. The process followed the PCC (Population, Concept, Context) (60, 61) and adhered to PRISMA-ScR (Preferred Reporting Items for Systematic Reviews and Meta-Analyses extension for Scoping Reviews) guidelines (62).

### 2.2 Inclusion/exclusion criteria

The inclusion and exclusion criteria were developed using the PCC framework (60, 61). Eligible studies including adolescents (aged 10–19 years) and young adults (20–30 years) with a formal diagnosis of BD based on standardized criteria (e.g., DSM-5, ICD-10). Consistency in developmental terminology, we use age groupings commonly applied in WHO/UN reporting, in which adolescents refer to individuals aged 10–19 years, and youth are often defined as those aged 15–24 years (with young people spanning 10–24 years) (63–65). For the purposes of this review, we extended the upper age limit to 30 years to avoid excluding studies in which young adults a subset of broader samples, or in which participants in early adulthood reported on experiences during recent adolescence (29, 63). Studies were excluded if participants had a primary diagnosis of psychotic disorders, current substance use disorders, intellectual disabilities, or a history of head trauma, brain injury, or neurosurgery, or if BD-specific outcomes were not reported. To be eligible, studies had to assess loneliness in individuals with BD; those not addressing loneliness in this context were excluded. Study designs considered were randomized or non-randomized trials and prospective or retrospective observational studies, while case reports, reviews, abstracts, editorials, opinion pieces, dissertations, and animal studies were excluded.

### 2.3 Study selection and data extraction

**Figure 1:**
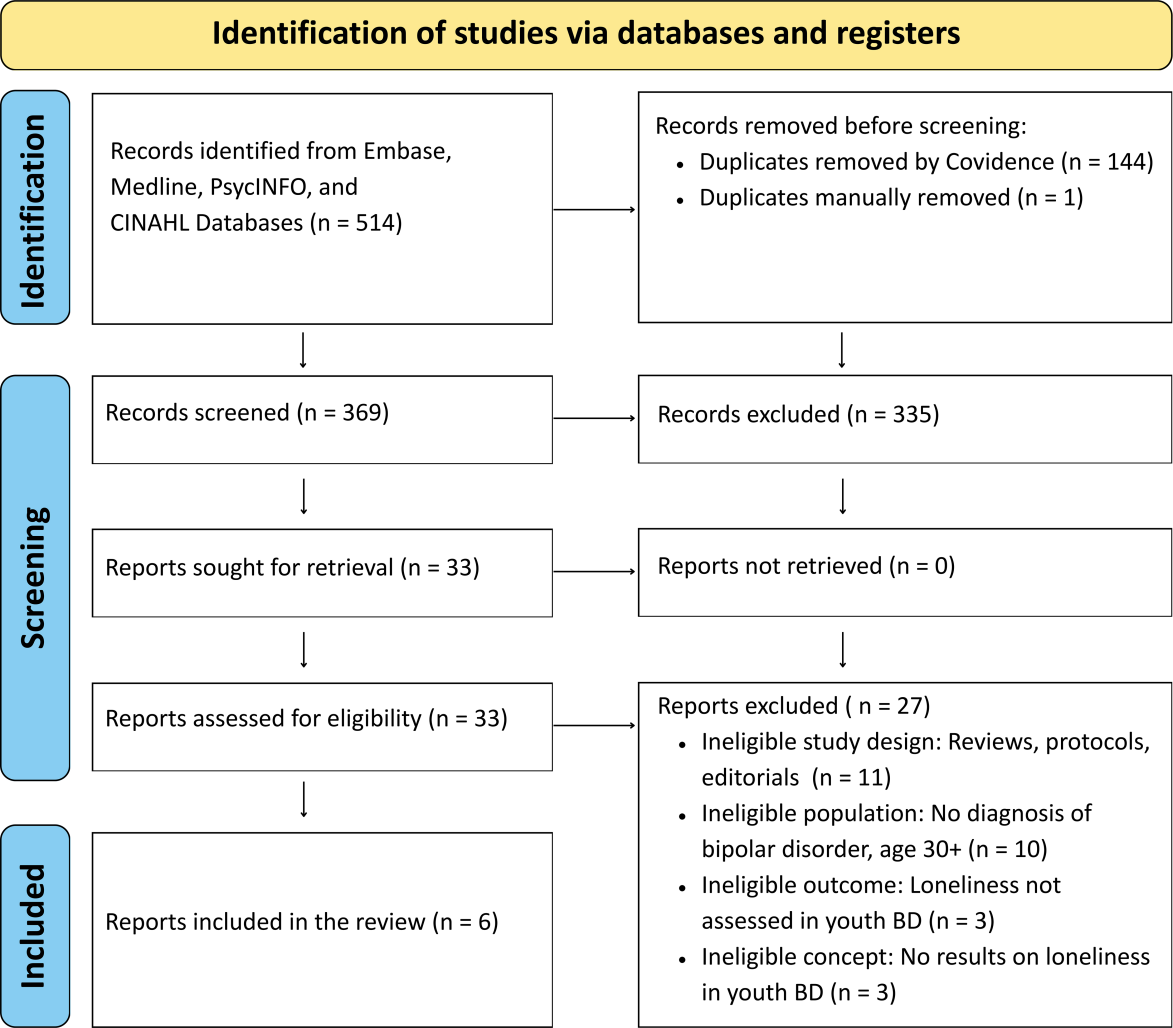
PRISMA search strategy diagram.

A total of 514 records were imported into Covidence for screening. Titles and abstracts were screened for eligibility by two independent reviewers (TT, SM), with conflicts resolved by a third reviewer (AD). Full texts of potentially relevant articles were assessed against the inclusion and exclusion criteria, resulting in six studies meeting eligibility criteria (**Figure 1**). For each included study, data were extracted on study design, setting, sample size, participant characteristics (e.g. sex, primary diagnosis), and measures of loneliness and/or social exclusion (**Table 1**).

**Table 1:** The characteristics of included studies.

Author, year, country	Study design	Sample size n (%)			Female, n (%)			Setting (outpatient; inpatient)	Assessment tools		Outcomes of interest
		BD	HC	N	BD	HC	N		Mood symptoms	Loneliness or social isolation^*^	
Wang et al., 2024, China (11)	Cross-sectional	246 (100)	–	246^a^	187 (76.0)	–	187 (76.0)	Outpatient	–	Loneliness; UCLA	Loneliness ranked 4th of 25 predictors; younger adolescents scored higher. ‘Feeling alone/lacking commonality’ predicted UD, while ‘feeling withdrawn’ predicted BD.
Roybal et al., 2022, USA (12)	Longitudinal	19 (57.5)	14 (42.4)	33	9 (27.2)	6 (18.1)	15 (45.4)	Outpatient	YMRS CDRS-R	Social isolation; Cyberball task	Youth with BD: greater left fusiform activation; HC: stronger fusiform–posterior cingulate/precuneus connectivity. BD patients relied on visual processing during social exclusion, whereas healthy peers integrated prior experiences.
Guo et al., 2021, China (10)	Cross-sectional	120 (100)	–	120	42 (35.0)	–	42 (35.0)	Outpatient, Inpatient	HAMD YMRS BPSS-R	Social isolation; BPSS-R	Social isolation (43.1%) was reported more often before the first depressive episode than before the first (hypo)manic episode (18.2%)
Smyth et al., 2020, USA (13)	Cross-sectional	8 (100)	–	8	–	–	–	Outpatient	–	Social isolation; Semi–structured interview	Participants reported persistent relational difficulties, compounded by depressive symptoms and poor self-regulation.
Nicholas et al., 2017, Australia (14)	Cross-sectional	89 (100)	–	89	77 (86.5)	–	77 (86.5)	Outpatient	PHQ-9 ASRM	Social isolation; Survey	Social isolation was reported by 98% as a coping strategy, but nearly half (49%) viewed it as unhelpful.
Heslin et al., 2015, UK (16)	Longitudinal	70 (15.1)	391 (84.8)	461^a,b^	37 (0.8)	230 (49.8)	267 (57.9)	Inpatient	–	Social isolation; MRCSDS	Social isolation was linked to BD; Individuals lacking close relationships/confidants were more than twice as likely to have BD (aOR = 2.03, CI = 1.16–2.57, p = 0.024).

^*^All loneliness and social isolation assessments were self-reported. ASRM, Altman Self-Rating Mania Scale; BD, bipolar disorder; BPSS-R, Bipolar Prodrome Symptom Interview and Scale–Retrospective; CDRS-R, Children’s Depression Rating Scale-Revised; HAM-D, Hamilton Depression Rating Scale; HC, healthy control; MRCSDS, Medical Research Council Socio-demographic Schedule; PHQ-9, Patient Health Questionnaire; UCLA, University of California Los Angeles Loneliness Scale; YMRS, Young Mania Rating Scale.

a sample also included subjects with major depressive disorder; sample also included individuals with schizophrenia.

## 3 Results

### 3.1 Study selection

The PRISMA review selection process for the articles included in the review is shown in the diagram in **Figure 1**. A total of 514 articles were generated from the search strategy from four databases after Covidence removed 145 duplicates. Using Covidence to screen for relevant articles from the title and abstract, 33 articles were selected. After the full-text review phase, six articles met the inclusion criteria and were eligible for inclusion in this review.

### 3.2 The characteristics of included studies

The six studies included in this review involved a total of 522 youth individuals with BD, with individual study sample sizes ranging from 8 (13) to 246 (11) for BD patients, and participants’ ages ranged from 14 (12) to 27 years (16). Five studies assessed social isolation as a proxy for loneliness (10, 12–14, 16), whereas only one measured loneliness using a validated scale (11). Two studies also included healthy controls (HC) (n = 405) (12, 16). Four studies were cross-sectional (10, 11, 13, 14) and five collected patient sex data (10–12, 14, 16), with predominantly female (64.7%) subjects. Three studies assessed mood symptoms using standardized rating scales (10, 12, 14). Participants were recruited from both inpatient (10, 16) and outpatient (10–14) settings. Geographically, the studies spanned multiple countries, including the United Kingdom (16), China (10, 11), USA (12, 13), and Australia (14). **Table 1** summarizes the characteristics of included studies.

### 3.3 Loneliness and social isolation in relation to diagnostic status in adolescents and youth with bipolar disorder

In a multi-center cross-sectional study of Chinese adolescents (aged 12–18) with unipolar depression (UD, n = 1547) and BD (n = 246), Wang et al., 2024 applied machine learning to distinguish UD from BD, including diverse sociodemographic (e.g., age, gender, romantic experience, smoking) and psychological (e.g., loneliness, self-esteem, parental bonding, alexithymia) parameters (11). Using a 25-item predictors shortlist their algorithm was able to discriminate between UD and BD with 80% accuracy and the most important features belonged to UCLA Loneliness Scale (58) and Parental Bonding Instrument (66). For instance, the 20-item UCLA evaluate the degree of loneliness, where higher total score indicates higher feelings of loneliness. Six out of the 25 predictors used in this study where UCLA items and the item 4 “I feel alone” was the second most important predictor. In adolescents aged 12–18 years, loneliness emerged as one of the higher-ranked contributors in a model discriminating UD from BD. Younger participants (12–15 years) reported higher loneliness scores than older adolescents, and exploratory item-level analyses suggested differential patterns: items reflecting feeling alone and lacking commonality were more strongly associated with UD, whereas items reflecting withdrawal were more strongly associated with BD (11).

Roybal et al., 2022 used cyberball, an fMRI-based social exclusion paradigm, to examine neural responses to social exclusion in adolescents aged 10–18 years with BD compared to HC (12). Youth with BD showed greater activation in the left fusiform gyrus (FFG) (p = 0.037), whereas HC exhibited stronger functional connectivity between the FFG and posterior cingulate/precuneus (p < 0.001) as well as the postcentral gyrus (p = 0.006). Within the BD group, subjective distress during exclusion was not significantly associated with connectivity of the FFG. Within the HC group, subjective distress during exclusion was not significantly associated with FFG connectivity. In contrast, within the HC group, greater subjective distress was associated with lower connectivity between the left FFG and the left posterior cerebellum (p = 0.004), and higher connectivity between the FFG and four regions: the left cuneus (p < 0.001), left precuneus (p = 0.008), right anterior insula (p = 0.001), and right premotor cortex (p = 0.001). These findings indicate potential group differences in neural activation during social isolation. Adolescents with BD showed relatively greater activation in regions linked to basic visual processing, whereas healthy peers engaged regions implicated in integrating social information and self-referential processing. The clinical significance of this pattern remains unclear, but it may be relevant to understanding loneliness-related experiences in youth with BD. The study found no significant group difference in subjective distress during social exclusion, as measured by the Need Threat Scale (p = 0.33). However, the BD group scored significantly higher than healthy controls on the anger [t(24) = 2.73, p = 0.012] and anxiety [t(28) = 2.15, p = 0.041] domains of the Rejection Sensitivity Questionnaire (RSQ) (67). As expected, the BD group also reported higher symptoms on the Young Mania Rating Scale (68) [t(21) = 3.04, p = 0.006] and the Children’s Depression Rating Scale–Revised [t(19) = 7.38, p < 0.001]. Within the BD group, subjective distress correlated with RSQ anger (ρ = –0.65, p = 0.012, q = 0.025) and showed a trend-level association with RSQ anxiety (ρ = 0.55, p = 0.041, q = 0.05), whereas no significant correlations were observed in controls. Overall, these exploratory findings suggest that neural response differences may co-occur with elevated rejection sensitivity in BD, warranting replication to clarify relevance to social-emotional functioning and loneliness.

Heslin et al. (2016) compared psychosocial risk factors including social isolation, across individuals with BD, schizophrenia, major depressive disorder, and healthy controls (16). Social isolation was operationalized using objective or structural indicators of social connectedness—relationship status (stable relationship vs. single), living arrangements (with others vs. alone), frequency of contact with friends and family, and the presence of close confidants—rather than subjective loneliness per se (16). In adjusted analyses, living alone, infrequent contact with friends (less than monthly), and unemployment were associated with a baseline diagnosis of BD. Being single and never having had a long-term relationship were associated with follow-up diagnosis of psychotic BD (aOR = 2.03, CI = 1.16–2.57, p = 0.014 and aOR = 2.08, CI = 1.04–4.16, p = 0.038, respectively) and schizophrenia (aOR = 5.36, CI = 3.46–8.28 and aOR = 4.08, CI = 2.51–6.63, respectively). Participants reporting no close confidants were also more likely to have BD (aOR = 2.03, CI = 1.16–2.57, p = 0.024) (16). Overall, structural indicators of reduced social connectedness were associated with BD and related diagnoses in this cohort, but they are not equivalent to subjective loneliness and do not support causal, predictive, or diagnostic conclusions.

### 3.4 Loneliness and social isolation as potential risk factors for mood episodes in adolescents and youth with bipolar disorder

In a cross-sectional retrospective study conducted in China (10) social isolation was assessed as a prodromal symptom among 120 youth with BD in clinical remission. Current mood symptoms, evaluated using the 17-item Hamilton Depression Rating Scale (HAMD-17) (69) and YMRS (68) did not significantly different between groups. Using the Bipolar Prodromal Symptoms Scale-Retrospective (BPSS-R (70);, the authors reported that social isolation was more frequently endorsed prior to a first depressive episode (43.1%) than prior to a first (hypo)manic (18.2%) episode (p = 0.003). However, no significant differences were observed in the reported duration or severity of social isolation across prodromal states. The prevalence of several prodromal symptoms also varied by episode type, including social isolation (p = 0.004), anxiety/nervousness (p = 0.009), and losing temper (p < 0.001). Social isolation, along anxiety/nervousness and indecisiveness reported more often prior to first depressive episodes than prior to first (hypo)manic episodes (p < 0.05), whereas symptoms such as anger, increased creativity, and giddy/clownish mood were more frequently reported prior to first (hypo)manic episodes (p < 0.05). These retrospective findings indicate differential endorsement of social isolation prior to depressive versus (hypo)manic onset, but do not establish predictive value or clarify how these reports relate to subjective loneliness.

Smyth et al., 2021 provided qualitative insights into social functioning and experiences of social isolation among eight young people with early-onset BD (EOBD; mean age = 21.75; SD = 2.31) (13). Participants described adolescence and early adulthood as periods of heightened social vulnerability, during which mood symptoms and self-regulation difficulties often coincided with challenges forming and sustaining relationships. They reported increasing avoidance coping and increasing social withdrawal, which they perceived as contributing to greater social isolation over time. As one participant reflected: “*Nobody understood what I was going through, even before the diagnosis … Nobody wanted to be near me*.” Participants linked symptom fluctuations with changes in social connectedness, describing that periods of worsening symptoms were often accompanied by withdrawal from social situations, interpersonal conflict, and heightened feelings of isolation. For some, isolation was framed as a self-protective “safe space” to manage distress, albeit with costs to family and peer relationships.

Consistent with prior research (71), participants reported that symptomatic periods were often accompanied by interpersonal strain and reduced perceived support (13). Difficulties forming and maintaining relationships, peer rejection, and self-regulation challenges were described alongside avoidance-based coping, including social withdrawal and selective disclosure of diagnosis (13). Collectively, participants viewed EOBD as a catalyst for interpersonal conflict, weakened support networks, and disrupted social development (13). Together, these accounts suggest that social isolation in youth with BD may co-occur with disrupted support networks and social development, though evidence remains limited and interpretive.

Nicholas et al. (2017) investigated self-management strategies among 89 youth with BD (mean age = 24) (14). Social isolation was reported by nearly all participants (98%) as a coping approach, alongside acceptance of diagnosis (98%) and engagement in psychoeducation (97%). However, almost half (49%) perceived isolation as unhelpful, contrasting with the perceived benefits of peer support and “stay-well” plans, which were used less frequently. Strategies most often rated as unhelpful included alcohol or other substance use (55%), social isolation (49%), and symptom monitoring (21%) (14). Consistent with prior evidence of interpersonal disruptions in BD (66, 67), participants described difficulties maintaining both friendships and romantic relationships. Overall, the findings suggests that while isolation is a common coping mechanism among youth with BD, it is often maladaptive and less effective than socially oriented approaches such as peer support and structured wellness planning (14).

## 4 Discussion

BD is a serious psychiatric condition affecting approximately 5% of adolescents worldwide (4, 9). Loneliness is increasingly recognized as a global epidemic linked to premature mortality, functional impairment, hospitalization, and future psychopathology (24, 27, 35, 42, 43, 54). Yet, there is limited understanding of how loneliness unfolds in the daily lives of adolescents and young adults at risk for, or diagnosed with BD. Overall, the included studies suggest higher levels of loneliness and/or social isolation in these populations compared with reference groups, although evidence remains limited and heterogeneous (10–14, 16).

Although evidence remains limited, loneliness and social isolation have been examined in relation to diagnostic status and adverse clinical or functional outcomes in adolescents with BD, with some studies suggesting they may help differentiate BD from other conditions (11, 12, 16) and may be associated with greater symptom burden or episode onset (10, 13, 14). A large study using a machine learning algorithm found that loneliness ranked among the top five predictors differentiating BD from unipolar depression in youth (11). Similarly, Guo et al. (2021) identified social isolation—alongside anxiety and indecisiveness—as a prominent symptom prior to the first mood episode, suggesting that loneliness may represent a prodromal marker in youth BD (10). Findings in adults converge with these observations. Loneliness has been associated with increased risk of mania onset, depression relapse, and suicidality, as well as with poorer global social functioning among general populations and people with depressive and bipolar disorders (18, 28). Studies indicate that even during euthymia, individuals with BD experience higher loneliness, reduced emotion recognition accuracy, and poorer social functioning than HCs (72). Loneliness was inversely related social functioning in both groups, and among BD patients, greater loneliness and lower emotion recognition accuracy were linked to deficits across social domains (72). Similarly, remitted BD patients reported higher loneliness and lower perceived social support than HCs, with loneliness-rather than social support- more strongly associated with current suicidal ideation, while perceived social support related more closely to lifetime suicidality (18).

Taken together, these findings suggest that loneliness may be more strongly associated with suicidal ideation, whereas social support may be more closely related to the transition from thoughts to suicidal behavior. They also indicate that a history of suicide attempts may be associate with diminished social networks, underscoring the potential value of interventions that rebuild and strengthen supportive relationships (17, 18, 72). Collectively, the available evidence points to a complex interplay among loneliness, emotion recognition, social functioning, and suicidality in BD, with social connectedness emerging as a potentially modifiable correlate. Future research should clarify the directionality of these associations and inform interventions designed to enhance interpersonal functioning and social skills.

Developmentally, peer and social interactions become increasingly salient across adolescence and are closely linked to emotional wellbeing, suggesting a rationale for considering interventions that strengthen connectedness and supportive relationships (73). Neuroimaging findings provide preliminary context for social-cognitive vulnerabilities in BD: during a social exclusion task, adolescents with BD showed greater activation in the left fusiform gyrus, whereas healthy controls demonstrated stronger connectivity with regions implicated in integrating prior social experiences (12). this pattern may reflect greater reliance on perceptual processing, rather than contextual appraisal, during exclusion in BD—a possibility that could be relevant to social-cognitive difficulties and mood dysregulation (12). Relatedly, adolescents with BD have been reported to misinterpret neutral facial expressions as threatening, which is broadly consistent with evidence describing heightened responsivity to emotional stimuli in BD (8).

At the neural level, loneliness has been associated with differences in reward-related responding within the ventral striatum, a region implicated in motivation, learning, and social reward processing (49, 50). Prior work suggests that, in non-lonely individuals, ventral striatal responses may be stronger to pleasant social cues than to non-social cues, whereas in lonelier individuals this pattern may be attenuated or reversed (57). Although the directionality and mechanisms remain uncertain, these findings are consistent with the possibility that loneliness relates to reduced neural sensitivity to positive social experiences, which could be relevant to social withdrawal or disengagement (49, 50). Taken together, these observations motivate further study of loneliness-related neural and social-cognitive correlates in BD, while emphasizing that current neuroimaging and machine-learning findings are preliminary and not yet clinically translatable.

Beyond neural mechanisms, social isolation and withdrawal recur across several included studies (11, 13, 16). Some youth with BD described withdrawal and acceptance-based strategies as part of symptom self-management, whereas socially oriented approaches (e.g., peer connection, stay-well planning) were reported less frequently (14). Stigma-related experiences, including stereotype endorsement, perceived discrimination, and low perceived control, were also associated with greater withdrawal among euthymic participants (72). In one cohort study, structural indicators of reduced social connectedness (e.g., fewer close relationships or confidants) were associated with BD diagnosis (16). Qualitative accounts similarly described strained friendships and family relationships, with selective disclosure and avoidance coping perceived as contributing to greater isolation (13). Overall, the limited evidence suggests that social isolation may co-occur with stigma, emotion-regulation difficulties, and relational strain in youth with BD; however, causal or diagnostic interpretations—and any assumption that isolation directly indexes subjective loneliness—remain premature. These findings support further work to clarify mechanisms and inform psychosocial interventions that strengthen social connectedness and promote adaptive coping.

### 4.1 Study limitations and strengths

This scoping review has several limitations that should temper interpretation. While well suited to mapping a small, heterogeneous evidence base, scoping reviews provide limited analytic depth because they typically do not include quantitative synthesis or formal quality/risk-of-bias appraisal. Accordingly, findings should be viewed as an overview to inform future systematic reviews and meta-analyses. Most included studies were cross-sectional designs (10, 11, 13, 14), which supports associations between loneliness/social isolation and BD-related outcomes, but cannot establish directionality; the observed relationships may reflect reciprocal process and shared determinants rather than an unidirectional “risk” pathway (10, 14, 18). Generalizability is further constrained by methodological features of the included studies: samples were generally small (limiting statistical power) and may have been affected by recruitment-related selection bias; age bands were often broad (combining adolescents and young adults) (10–14, 16), and sex distributions were skewed toward female participants, limiting developmental and sex-stratified inferences (19, 45, 72). Several studies provided limited characterization of clinical context, including incomplete reporting or consideration of psychiatric/medical comorbidities and key sources of confounding—such as current mood state, comorbid anxiety, and medication status (45, 72–74)—all of which could influence both loneliness and functioning (38, 52, 54).

Measurement approaches also varied and commonly relied on self-report, sometimes using single-item or non-validated assessments of loneliness, social isolation, and/or mood symptoms, which may inflate associations via shared method variance and sensitivity to transient affective states (10–12). In addition, included studies spanned different countries and settings, raising the possibility that cultural norms and healthcare-system differences (e.g., access to psychosocial supports, service pathways, stigma context) shape how loneliness/social isolation are experienced, reported, and addressed. Despite these constraints, the review has notable strengths: it followed JBI scoping review guidance, applied a comprehensive multi-database search without language or date restrictions supplemented by hand-searching (59)., and intentionally included both “loneliness” and “social isolation” terminology to capture a broader set of relevant evidence in a field where these constructs are frequently conflated but conceptually distinct (23).

To contextualize these mapped associations within a broader framework and identify testable questions, the next section proposes an integrative, hypothesis-generating biopsychosocial model linking BD with loneliness/social isolation in youth; this model extends beyond the direct evidence of the included studies and is presented to guide future longitudinal and mechanistic research rather than to summarize established causal pathways.

### 4.2 Integrative biopsychosocial model linking BD with loneliness/social isolation in youth

**Figure 2:**
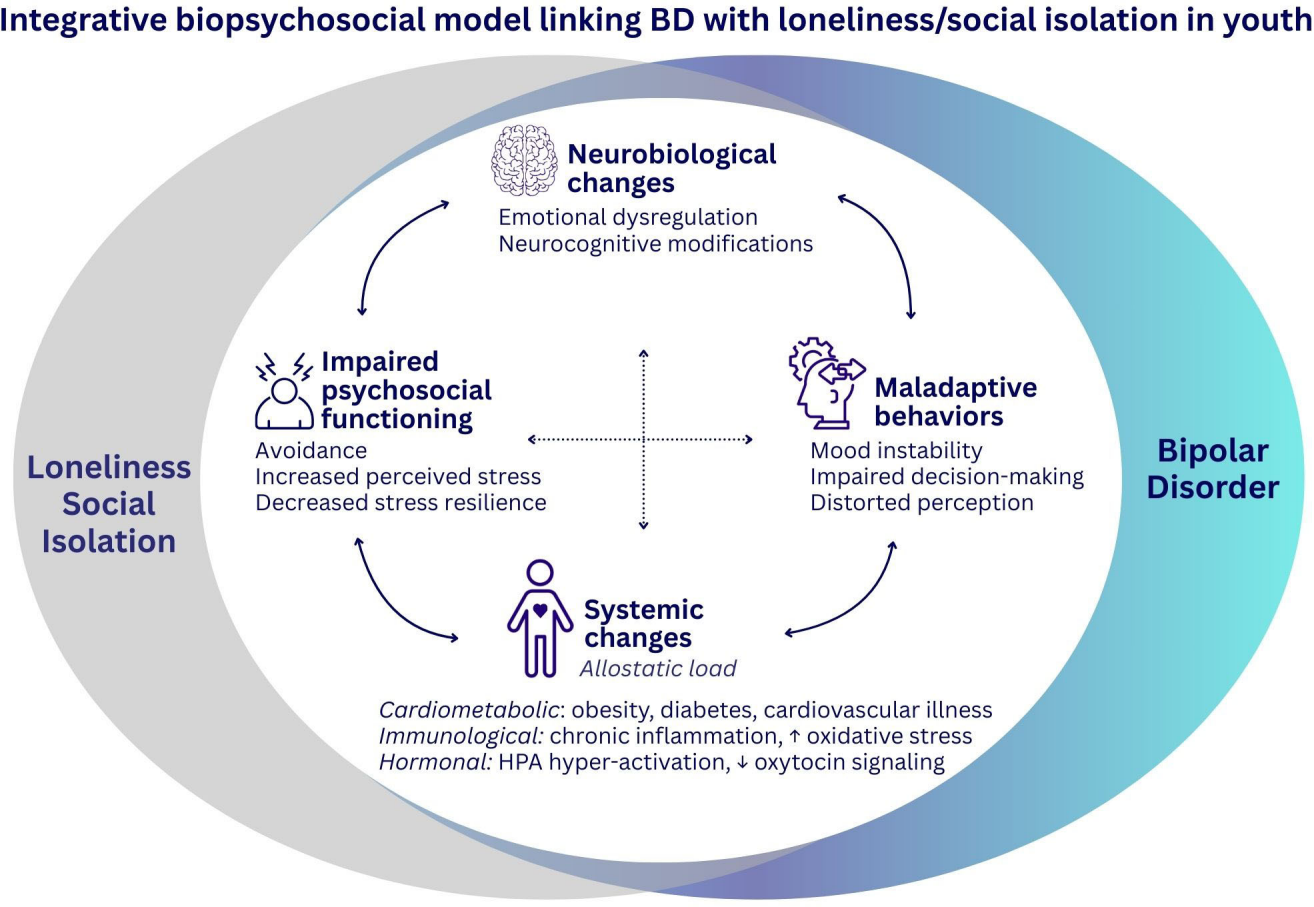
Integrative biopsychosocial model linking BD with loneliness/social isolation in youth.

The increased premature mortality observed in BD is partly attributed to aging-related diseases (75, 76), such as dementia, cardiovascular illness, and metabolic syndrome, which involve hypertension, impaired glucose metabolism, obesity, and dyslipidemia—factors that elevate the risk for type 2 diabetes and cardiovascular-related death (3, 77). These risks are further compounded by systemic abnormalities such as elevated stress hormones (e.g., cortisol, epinephrine), proinflammatory markers (e.g., C-reactive protein, IL-6, TNF-α), and oxidative stress (44, 78–80). Arising from the body’s attempts to adapt to repeated or chronic stress, these biological changes—often described as allostatic load—exert damaging effects on the brain and other organs (78, 81). In turn, they accelerate illness progression through disruptions in neural circuits, with consequences spanning behavioral (e.g., mood instability, emotional dysregulation) (2, 82), systemic (e.g., obesity, chronic inflammation, cardiometabolic disease) (3, 83), and functional domains (e.g., cognitive decline) (5, 6, 82). **Figure 2** illustrates biopsychosocial model linking BD with loneliness/social isolation in youth.

Similarly to BD, loneliness and social isolation activates biological and cognitive processes that heighten vulnerability to mood instability, emotion dysregulation, and suicidality (19, 50, 84), thereby contributing to illness severity (**Figure 2**). For instance, prolonged activation of the hypothalamic–pituitary–adrenal (HPA) axis elevates stress hormone release, a pathway strongly linked to suicide risk in BD (28, 37, 44). Chronic stress also impairs emotion regulation and alters brain circuitry (e.g., dopaminergic pathways) predisposing individuals to mood instability (50, 85). These neurocognitive changes often manifest as anhedonia, hopelessness, and apathy, reinforcing social isolation (50, 52, 72). Cognitive biases may intensify this cycle, as individuals with high trait loneliness perceive the social world as threatening and preferentially attend to negative stimuli (25, 84); **Figure 2**. Altogether, these findings are consistent with the hypothesis that loneliness and social isolation may contribute to greater allostatic load in youth with BD and be associated with increased mental and physical health risks, with potential implications for illness progression.

Understanding how loneliness and social isolation relate to affective instability and functioning in adolescents and youth with BD may help to inform interventions that address biological, emotional, and cognitive pathways. Lifestyle strategies (e.g., balanced nutrition and regular physical activity) can reduce stress and inflammation and may help mitigate cardiometabolic vulnerability linked to allostatic load (38, 53, 54). Psychological interventions—including psychoeducation, mindfulness, and cognitive–behavioral approaches—may reduce maladaptive appraisals and attentional biases that can maintain perceived loneliness (5, 52). At a biological level, emerging pharmacological approaches (e.g., allopregnanolone) have been proposed to modulate HPA axis activity and stress reactivity (86). Collectively, these approaches may help interrupt reinforcing cycles in which loneliness co-occurs with heightened stress physiology, altered reward/salience processing, and negative cognitive biases—processes that can coincide with emotional dysregulation and functional impairment. Although loneliness is not a formal diagnostic criterion, its association with poorer daily functioning (48, 50, 52, 55), supports considering it a potential modifiable therapeutic target in BD and related conditions.

### 4.3 Future clinical and research avenues on loneliness and social isolation in BD and other mental health conditions

Future research would benefit from longitudinal designs, validated and standardized measures of loneliness/social isolation, and greater attention to biopsychosocial mechanisms that may link these constructs with BD-related outcomes. Addressing current methodological limitations may clarify how loneliness and social isolation relate to symptom trajectories and functioning over time, and support the development and testing of targeted, evidence-based interventions for youth with BD.

Assessment approaches should integrate self-report with objective indicators (e.g., social network characteristics, interaction frequency, or digitally derived markers) to better capture the multidimensional nature of social connection (22, 23, 57). Although loneliness is not a formal diagnostic criterion, its repeated association with functional impairment in the available literature suggests it may warrant consideration as a potential modifiable intervention target in BD and other mental disorders pending prospective confirmation.

Digital technologies may also help detect early signals of increasing loneliness (e.g., shifts in sleep, activity, or mood patterns) and support timely outreach (83, 87). At the same time, social media may offer connection opportunities while also amplifying distress or symptom vulnerability for some youth with or at-risk for BD and related conditions (88, 89). As policies and regulations around technology use evolve, mental health professionals can contribute to guidance that balances access to supportive digital spaces with safeguards for vulnerable users.

## 5 Conclusion

To our knowledge, this is the first scoping review to synthesize evidence on loneliness and social isolation among adolescents and young people with BD. Although loneliness is not a formal diagnostic feature, the available studies suggest it is commonly reported and meaningfully associated with psychosocial functioning and illness-relevant outcomes in this population. However, the current evidence base is limited and largely correlational and does not yet establish whether loneliness functions as a risk factor, diagnostic differentiator, or consequence of illness processes. Future work should prioritize longitudinal and mechanistic designs to clarify temporal relationships and to test how loneliness and perceived social isolation relate to mood symptoms, cognition, and biological stress systems. Integrating measures of social functioning and candidate indicators of allostatic load may help characterize clinically relevant subgroups and inform the development and evaluation of targeted, accessible interventions. Clinically, routine assessment of loneliness and social isolation as part of comprehensive evaluations may help identify unmet needs and guide person-centered care; scalable evidence-based supports—delivered in person or digitally—should be tested for their ability to improve social connectedness and functioning in youth with BD.
