# Supplementary material for: Loneliness in adolescents and youth with bipolar disorder: a scoping review
Source: Front Psychiatry. 2026 Mar 13;17:1696515. doi: 10.3389/fpsyt.2026.1696515 (PMC13021885; doi:10.3389/fpsyt.2026.1696515)
Supplement: Supplementary file 1 [file Table1.docx]

**Search strategies**

**Ovid MEDLINE(R) ALL <1946 to June 27, 2025>**

1 adolescent/ or young adult/ 2869899

2 (youth* or adolescen* or young adult* or teenager*).tw,kf. 605481

3 (young men or young women or young people).tw,kf. 87122

4 ((college or university) adj2 age*).tw,kf. 5318

5 student*.tw,kf. 415981

6 Child/ 2027380

7 (child or children or youngster* or p?ediatric*).tw,kf. 1860903

8 or/1-7 4934934

9 "bipolar and related disorders"/ or bipolar disorder/ 47530

10 (bipolar or affective disorder*).tw,kf. 96286

11 8 and 10 19466

12 Loneliness/ 8212

13 Social Isolation/ 17530

14 (loneliness or isolation).tw,kf. 333638

15 12 or 13 or 14 343752

16 11 and 15 128

17 exp animals/ not humans/ 5351726

18 16 not 17 122

**Embase Classic+Embase <1947 to 2025 June 27>**

1 *adolescent/ 43969

2 *young adult/ 2917

3 (youth* or adolescen* or young adult* or teenager*).tw. 784850

4 (young men or young women or young people).tw. 123940

5 ((college or university) adj2 age*).tw. 6798

6 student*.tw. 552315

7 *child/ 176597

8 (child or children or youngster* or p?ediatric*).tw. 2628193

9 or/1-8 3669363

10 exp bipolar disorder/ 105683

11 (bipolar or affective disorder*).tw. 142108

12 10 or 11 177572

13 9 and 12 19461

14 loneliness/ 19406

15 social isolation/ or social alienation/ 40346

16 (loneliness or isolation).tw. 424737

17 or/14-16 449460

18 13 and 17 239

19 (exp animal/ or nonhuman/ or animal experiment/) not exp human/ 8704015

20 18 not 19 228

**APA PsycInfo <1806 to June 2025 Week 4>**

1 (youth* or adolescen* or young adult* or teenager*).mp. 731265

2 (young men or young women or young people).mp. 55170

3 ((college or university) adj2 age*).mp. 9081

4 ((university or college) adj2 student*).mp. or student*.tw. 724143

5 (child or children or youngster* or p?ediatric*).mp. 881582

6 or/1-5 1908772

7 exp bipolar disorder/ 37054

8 (bipolar or affective disorder*).tw. 63803

9 7 or 8 68442

10 6 and 9 17683

11 loneliness/ 8925

12 social isolation/ or social exclusion/ 12224

13 (loneliness or isolation).tw. 55065

14 11 or 12 or 13 60278

15 10 and 14 117

**Cinahl Ultimate <2025-06-30>**

| **#** | **Query** | **Limiters/Expanders** | **Last Run Via** | **Results** |
| --- | --- | --- | --- | --- |
| S1 | (MH "Bipolar Disorder+") | Search modes - Proximity | Interface - EBSCOhost Research Databases Search Screen - Advanced Search Database - CINAHL Ultimate | 14,097 |
| S2 | XB ((bipolar or affective disorder*)) | Search modes - Proximity | Interface - EBSCOhost Research Databases Search Screen - Advanced Search Database - CINAHL Ultimate | 21,121 |
| S3 | S1 OR S2 | Search modes - Proximity | Interface - EBSCOhost Research Databases Search Screen - Advanced Search Database - CINAHL Ultimate | 25,478 |
| S4 | (MH "Loneliness") | Search modes - Proximity | Interface - EBSCOhost Research Databases Search Screen - Advanced Search Database - CINAHL Ultimate | 8,853 |
| S5 | (MH "Social Isolation") OR (MH "Social Alienation") | Search modes - Proximity | Interface - EBSCOhost Research Databases Search Screen - Advanced Search Database - CINAHL Ultimate | 14,430 |
| S6 | XB ((loneliness or isolation)) | Search modes - Proximity | Interface - EBSCOhost Research Databases Search Screen - Advanced Search Database - CINAHL Ultimate | 41,954 |
| S7 | S4 OR S5 OR S6 | Search modes - Proximity | Interface - EBSCOhost Research Databases Search Screen - Advanced Search Database - CINAHL Ultimate | 52,741 |
| S8 | S3 AND S7 | Search modes - Proximity | Interface - EBSCOhost Research Databases Search Screen - Advanced Search Database - CINAHL Ultimate | 258 |
| S9 | (MH "Child") OR (MH "Adolescence") | Search modes - Proximity | Interface - EBSCOhost Research Databases Search Screen - Advanced Search Database - CINAHL Ultimate | 944,239 |
| S10 | (MH "Young Adult") | Search modes - Proximity | Interface - EBSCOhost Research Databases Search Screen - Advanced Search Database - CINAHL Ultimate | 298,552 |
| S11 | XB ((youth* or adolescen* or young adult* or teenager*)) | Search modes - Proximity | Interface - EBSCOhost Research Databases Search Screen - Advanced Search Database - CINAHL Ultimate | 275,573 |
| S12 | XB ((young men or young women or young people).) | Search modes - Proximity | Interface - EBSCOhost Research Databases Search Screen - Advanced Search Database - CINAHL Ultimate | 46,081 |
| S13 | XB (((college or university) N2 age*)) | Search modes - Proximity | Interface - EBSCOhost Research Databases Search Screen - Advanced Search Database - CINAHL Ultimate | 3,780 |
| S14 | XB (student*) | Search modes - Proximity | Interface - EBSCOhost Research Databases Search Screen - Advanced Search Database - CINAHL Ultimate | 230,212 |
| S15 | XB ((child or children or youngster*)) | Search modes - Proximity | Interface - EBSCOhost Research Databases Search Screen - Advanced Search Database - CINAHL Ultimate | 564,849 |
| S16 | XB (pediatric* or paediatric*) | Search modes - Proximity | Interface - EBSCOhost Research Databases Search Screen - Advanced Search Database - CINAHL Ultimate | 188,862 |
| S17 | S9 OR S10 OR S11 OR S12 OR S13 OR S14 OR S15 OR S16 | Search modes - Proximity | Interface - EBSCOhost Research Databases Search Screen - Advanced Search Database - CINAHL Ultimate | 1,591,892 |
| S18 | S8 AND S17 | Search modes - Proximity | Interface - EBSCOhost Research Databases Search Screen - Advanced Search Database - CINAHL Ultimate | 47 |
